# Gelatin–chlorin e6 conjugate for in vivo photodynamic therapy

**DOI:** 10.1186/s12951-019-0475-1

**Published:** 2019-04-05

**Authors:** Jihwan Son, Gawon Yi, Moon-Hwa Kwak, Seung Mok Yang, Jae Myung Park, Bo-In Lee, Myung-Gyu Choi, Heebeom Koo

**Affiliations:** 1 Department of Medical Life Sciences, College of Medicine, The Catholic University of Korea, 222 Banpo-daero, Seocho-gu, Seoul, 06591 Republic of Korea; 2 Department of Biomedicine & Health Sciences, College of Medicine, The Catholic University of Korea, 222 Banpo-daero, Seocho-gu, Seoul, 06591 Republic of Korea; 3 Division of Gastroenterology, Department of Internal Medicine, Seoul St. Mary’s Hospital, The Catholic University of Korea, Seoul, Republic of Korea; 4 Catholic Photomedicine Research Institute, College of Medicine, The Catholic University of Korea, Seoul, Republic of Korea

**Keywords:** Gelatin, Photodynamic therapy, Nanoparticle, Chlorin e6, Drug delivery, Tumor-targeting

## Abstract

**Background:**

Improving the water solubility of hydrophobic photosensitizer and increasing its accumulation in tumor tissue are essential for in vivo photodynamic therapy (PDT). Considering commercialization or clinical application in future, it will be promising to achieve these purposes by developing new agents with simple and non-toxic structure.

**Results:**

We conjugated multiple chlorin e6 (Ce6) molecules to gelatin polymer, synthesizing two types of gelatin–Ce6 conjugates with different amounts of Ce6: gelatin–Ce6-2 and gelatin–Ce6-8. The resulting conjugates remained soluble in aqueous solutions for a longer time than hydrophobic Ce6. The conjugates could generate singlet oxygen and kill tumor cells upon laser irradiation. After intravenous injection into SCC-7 tumor-bearing mice, gelatin–Ce6-2 showed prolonged blood circulation and highly increased accumulation in tumor tissue as observed in real-time imaging in vivo. After laser irradiation, gelatin–Ce6-2 suppressed tumor growth completely and enabled improved PDT compared to free Ce6 and gelatin–Ce6-8.

**Conclusions:**

This work demonstrates that a simple structure based on photosensitizer and gelatin can highly improve water solubility and stability. Superior tumor tissue accumulation and increased therapeutic efficacy of gelatin–Ce6 during in vivo PDT showed its high potential for clinical application.
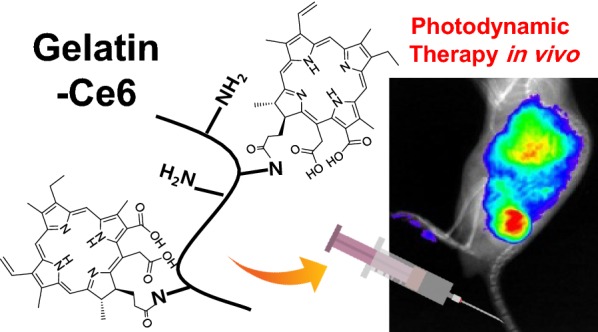

**Electronic supplementary material:**

The online version of this article (10.1186/s12951-019-0475-1) contains supplementary material, which is available to authorized users.

## Background

Photodynamic therapy (PDT) using photosensitizer is an effective treatment for various cancers [1]. Photosensitizer is a special kind of dye that is not toxic in the absence of light. However, irradiating photosensitizer with light of the appropriate wavelength generates cytotoxic singlet oxygen and can destroy cancer cells [2]. Based on this mechanism, PDT has been used to treat colorectal, intraperitoneal, head and neck, prostate, and breast cancers [3, 4]. Notably, photosensitizer can generate singlet oxygen and fluorescence simultaneously, which makes it useful for both imaging and therapy [3, 5–7]. However, most types of photosensitizer have poor water solubility due to their multi-ring chemical structures. Furthermore, low selectivity of photosensitizer for diseased tissue and cells after intravenous injection impairs the effectiveness of PDT in clinical practice.

To overcome these limitations, researchers have tried various methods [8, 9]. One method involves conjugating hydrophobic photosensitizer to a hydrophilic polymer [10]. Unlike other chemical drugs, photosensitizer does not lose its therapeutic ability when conjugated to another molecule such as a polymer [11]. In particular, tumor tissue accumulation of macromolecule drugs in nano-scale is higher than that of small molecules through enhanced permeation and retention (EPR) [12, 13]. These macromolecules remain in circulation longer and pass through fenestrated vessels in sites of angiogenic disease such as tumors. In addition, macromolecules that have accumulated in tumor tissue are not removed rapidly due to inefficient lymphatic drainage of the tumor. For this purpose, it is important to select a suitable polymer and to optimize the resulting chemical structure of the conjugates [14, 15].

Herein, we conjugated a hydrophobic photosensitizer, chlorin e6 (Ce6), to a gelatin biopolymer for efficient photosensitizer delivery and PDT in vivo. Gelatin is one of the biopolymers obtained from thermal treatment of collagen [16]. It is hydrophilic and non-toxic, so it is widely used in foods and biomedical applications [17]. Moreover, in different with other natural polysaccharides like hyaluronic acid, chondroitin sulfate, heparin, and pullulan, gelatin has multiple amine groups. They enable facile modification of gelatin with other molecules by chemical conjugation. Ce6 has been selected as a photosensitizer because it has high efficiency in singlet oxygen generation, and it is activated by near-infrared wavelengths, which is suitable for penetration into deep tissues [18]. We conjugated varying amounts of Ce6 to gelatin, and the products’ characteristics were analyzed both in vitro and in vivo. After synthesis of these gelatin–Ce6 nano-conjugates, we tested their stability in aqueous conditions and their singlet oxygen generation efficiency. Murine squamous carcinoma (SCC-7) cells were used to evaluate cellular uptake, cytotoxicity, and phototoxicity. After intravenous injection of gelatin Ce6 into mice bearing SCC-7 tumors, we analyzed the compound’s real-time biodistribution and tumor tissue accumulation via an in vivo optical imaging system [19]. Finally, the therapeutic results of gelatin–Ce6 were obtained and analyzed after PDT in the same mice.

## Methods

### Materials

Ce6 was purchased from Frontier Scientific, Inc. (Logan, UT, USA). *N*-Hydroxysuccinimide (NHS), *N*,*N*-Diisopropylethylamine (DIPEA), Eosin Y solution, and Mayer’s Hematoxylin Solution for H&E staining were purchased from Sigma-Aldrich (St. Louis, MO, USA). Triton X-100 and dimethyl sulfoxide (DMSO) were purchased from Samchun Chemical Co., Ltd. (Seoul, Gangnam-gu, Korea). Xylene and ethyl alcohol, 99.9% were purchased from Duksan (Seongnam, Gyeonggi-do, Korea). Dialysis membrane (MWCO:25 kD) was purchased from Spectrum Lab, Inc. (Rancho Dominguez, CA, USA). Four percent paraformaldehyde solution and thiazolyl blue tetrazolium bromide (3-[4,5-dimethyl-thiazol-2-yl]-2,5-diphenyltetrazolium bromide: MTT) were purchased from Biosesang (Seongnam, Gyeonggi-do, Korea). Optimal cutting temperature (O.C.T.) compound was purchased from Sakura Finetek (Tokyo, Japan). 1-Ethyl-3-(3-dimethylaminopropyl)carbodiimide hydrochloride (EDC), 4′,6-diamidine-2′-phenylindole dihydrochloride (DAPI), Hoechst 33342, Singlet Oxygen Sensor Green Reagent (SOSG), and phosphate buffered saline (PBS, pH 7.4) were purchased from Thermo Fisher Scientific (Waltham, MA, USA). Type B gelatin (220 Bloom, MW = 40–50 kDa) was obtained from Sammi Industrial Co., Ltd. (Ansan, Gyeonggi Do, Korea). Roswell Park Memorial Institute (RPMI) medium and fetal bovine serum (FBS) were purchased from Biowest (Nuaille, France). Antibiotic–antimycotic solution was purchased from Gibco BRL (Grand Island, NY, USA).

### Synthesis of gelatin–Ce6

The carboxyl group of Ce6 was directly conjugated with the amine groups of gelatin polymers by amide coupling. Briefly, 3 mL of 1.2 mg (2.0 μmol) or 4.8 mg (8.0 μmol) Ce6 solution (DMSO:water = 1:1) was prepared and mixed with 1.9 mg EDC, 1.2 mg NHS, and 3.5 μL DIPEA. Then, 5.0 mg of gelatin was added to the resulting solution, and the mixture was stirred at room temperature overnight. To remove the remaining free Ce6, the mixture was dialyzed sequentially with 50% ethanol, 100% ethanol, and deionized water for 1 day. Then, the product was lyophilized to obtain a dark powder.

### Characterization of gelatin–Ce6

The amount of Ce6 conjugated to gelatin was analyzed by measuring the fluorescence of Ce6 (λ_ex_/λ_em_ = 405/650 nm) using a synergy H1 Hybrid Multi-Mode Reader (BioTek Instruments, Inc., Winooski, VT, USA). A detergent solution (1% Triton X-100, DMSO:PBS:DW = 5:4:1) was used to solubilize Ce6 and gelatin–Ce6 completely for measurement. The size of prepared gelatin–Ce6-2 and gelatin–Ce6-8 were measured in PBS (pH 7.4) using a nanoparticle analyzer (SZ-100Z; Horiba, Japan) and 21 CFR Part 11 software. The shape and size of them were also observed by transmission electron microscopy (TEM) after a negative staining with 2% (w/v) uranyl acetate solution. To test the stability in aqueous solution, free Ce6, gelatin–Ce6-2, and gelatin–Ce6-8 were dispersed in PBS at 10 mg/mL (Ce6 concentration), placed in a tube, and left at room temperature for 1 month.

### Singlet oxygen generation from gelatin–Ce6

Singlet oxygen generation was analyzed using SOSG. Each sample (free Ce6, gelatin–Ce6-2, or gelatin–Ce6-8) was added to SOSG (0.1 μM in PBS) containing 1% DMSO for a final 2 μM Ce6 concentration. Then, the samples were irradiated with a 658 nm laser (0.3 W) for different time periods (0.5, 1, 2, 4, 9, 12, or 15 min). SOSG fluorescence (λ_ex_/λ_em_ = 488/525 nm) was monitored with a Synergy H1 Hybrid Multi-Mode Reader (BioTek Instruments, Inc., VT, USA).

### Cellular uptake of gelatin–Ce6

SCC-7 cell line (a mouse squamous cell carcinoma line) was obtained from the American Type Culture Collection (Rockville, MD) and was cultured in RPMI medium with 10% FBS and 1% Antibiotic–Antimycotic at 37  °C in a humidified atmosphere of 5% CO_2_. For the cellular uptake test, SCC-7 cells were seeded into 24-well plates at a density of 4 × 10^4^ cells/well and grown for 1 day. Then, the medium was replaced with 500 μL of serum-free medium containing free Ce6 (1 to 16 μM) or gelatin–Ce6 (1 to 16 μM of Ce6), and the cells were incubated for 120 min. Subsequently, the cells were treated with Hoechst 33342 and washed with PBS. The medium was replaced with 100 μL of RPMI without phenol red, and imaging was performed with a Fluorescence Inverted Microscope IX71 (Olympus, Tokyo, Japan). Quantitative analysis of cellular uptake was performed by flow cytometry (FACS Canto II, BD Biosciences, Bedford, MA, USA). SCC-7 cells were seeded in a T-75 cell culture flask (SPL Life Sciences, Pocheon-si, Gyeonggi-do, Korea) at a density of 5 × 10^5^ cells/plate and cultured at 37 °C for 48 h. After reaching 80% confluence, the medium was replaced with 15 mL of serum-free medium containing free Ce6 (0.2 μM) or gelatin–Ce6 (0.2 μM based on the free Ce6 amount), and the cells were incubated for 2 h. Then, the cells were washed with PBS and trypsinized. The cells were harvested at 1500 rpm for 5 min and the pellets were washed twice with 1 mL of PBS. They were then dispersed in 1 mL PBS with 1% BSA and transferred to a 5 mL polystyrene round bottom tube (Corning, NY, USA). For each sample, 10,000 events were collected and analyzed.

### Laser-induced phototoxicity of gelatin–Ce6 in cell viability assay

The viability of SCC-7 cells was determined by MTT assay. SCC-7 cells were seeded into a 96-well plate at a density of 2 × 10^4^ cells/well and grown for 1 day. After removing the medium and washing with PBS, the cells were treated with different concentrations of free Ce6 or gelatin–Ce6 in 100 μL culture medium (0, 1, 2, 4, 8, or 16 μM) for 30 min or 2 h. Then, the wells were washed with PBS, and 100 μL of phenol red-free medium was added to each well. To test toxicity in the dark, the samples were analyzed directly by MTT assay. For the phototoxicity test, each well was laser irradiated (658 nm, 0.3 W, 0.75 J). A 20 µL aliquot of MTT solution (0.5 mg/mL) was then added to each well, followed by incubation for 4 h. Then, the medium was removed, and 100 µL of DMSO was added. The absorbance of MTT was measured with a PowerWave HT Microplate Spectrophotometer (BioTek Instruments, Inc., VT, USA) at 570 nm.

### In vivo and ex vivo imaging of mice

To establish the tumor model, 1 × 10^6^ SCC-7 cells (80 μL) were injected into the left femoral regions of 4-week-old C3H/HeN male mice. After the tumors grew to an average volume of 150  ±  30 mm^3^, a saline solution of free Ce6, gelatin–Ce6-2, or gelatin–Ce6-8 was injected via the tail vein at a concentration of 2.5 mg/kg (based on Ce6) (n = 3). Whole body fluorescence images were obtained 1, 3, 6, 12, and 24 h post injection by IVIS Lumina XRMS (PerkinElmer, Inc, Waltham, MA, USA) equipped with a Cy5.5 filter (excitation 660 nm, emission 710 nm). Images were analyzed using Living Image 4.5 software (PerkinElmer Inc, Waltham, MA, USA).

Also, we collected blood samples of approximately 10 µL from the eyes of the mice at different time points (1, 3, 6, 12, and 24 h), using a heparin-coated capillary tube. The blood samples were mixed with detergent solution (1% Triton X-100, DMSO:PBS:DW = 5:4:1) at a ratio of 1:9, and the fluorescence intensity of Ce6 was measured by IVIS Lumina XRMS. After the mice were euthanized 24 h post-injection, tumors and other organs (heart, lung, liver, spleen, and kidney) were removed and analyzed by IVIS Lumina XRMS.

After ex vivo imaging, tumors extracted from the mice were placed in molds with O. C. T. compound and frozen at − 80 °C. Then, they were cut into slices with a thickness of 6 μm, washed 3 times for 4 min each with PBS, and treated with DAPI. Fluorescence images of the sliced tumor tissues were obtained using a Fluorescence Inverted Microscope IX71 (Olympus, Tokyo, Japan).

To observe clearance route of gelatin–Ce6-2 and gelatin–Ce6-8, they we intravenously injected into CD1 mice, which were euthanized at 3, 6, 12, and 24 h post injection and performed a surgery to open abdomen. Then, whole body fluorescence images were obtained by IVIS Lumina XRMS with same condition. Also, 10 μL of blood samples from each mouse were collected. Collected blood samples were analyzed using IVIS Lumina XRMS at just and 3 h after mixed with 90 μL PBS (pH 7.4). The major organs (heart, lung, liver, spleen, and kidney) were dissected and obtained ex vivo images by IVIS Lumina XRMS. Fifty milligrams of dissected organs tissue were washed with PBS (pH 7.4) and grinded with tissue grinder in 100 μL of Mammalian protein extract buffer solution. Then, grinded organ tissues were dispersed by a C505 probe sonicator (Sonics; Newtown, CT, USA) for few seconds. A 100 μL of detergent solution (1% Triton X-100, DMSO:PBS:DW = 5:4:1) was added to each sample (100 μL) and analyzed using IVIS Lumina XRMS.

### In vivo photodynamic therapy

SCC-7 tumor-bearing mice were prepared similarly to those in the in vivo imaging study. A free Ce6, gelatin–Ce6 2 μM, or gelatin–Ce6 8 μM solution, each containing 2.5 mg/kg of Ce6, was injected into the tail vein (n = 4) when the tumor volume reached approximately 150 ± 30 mm^3^. At 4 and 12 h after the sample injection, the tumor site of each mouse was irradiated with a red laser (658 nm, 0.3 W, 200 J). Then, the tumor sizes of all mice were monitored over 14 days using a caliper and calculated based on the formula (L × S^2^ × 1/2), where L and S indicate the longer and shorter tumor diameters, respectively. According to the animal protection act of guideline for IACUC (Institutional Animal Care and Use Committee, Animal and Plant Quarantine Agency, Korea), when the diameter of subcutaneous tumor was 17 mm or more, the experiment was stopped, and euthanasia was performed.

For H&E staining, tumor tissue samples from the 4 groups of mice and major organs from gelatin–Ce6 treated mice were collected. The samples were fixed with 10% formalin solution and embedded in paraffin blocks. The resulting blocks were sectioned into 4-µm-thick slices and stained with H&E. Histopathological profiles were observed using a fluorescence microscope (AX70, TR-62A02, Olympus, Tokyo, Japan).

### Statistics

The statistical significance of differences between groups was analyzed using one-way ANOVA. p values below 0.01 were considered significant.

## Results and discussion

### Synthesis and characterization of gelatin–chlorin e6 conjugate (gelatin–Ce6)

**Fig. 1:**
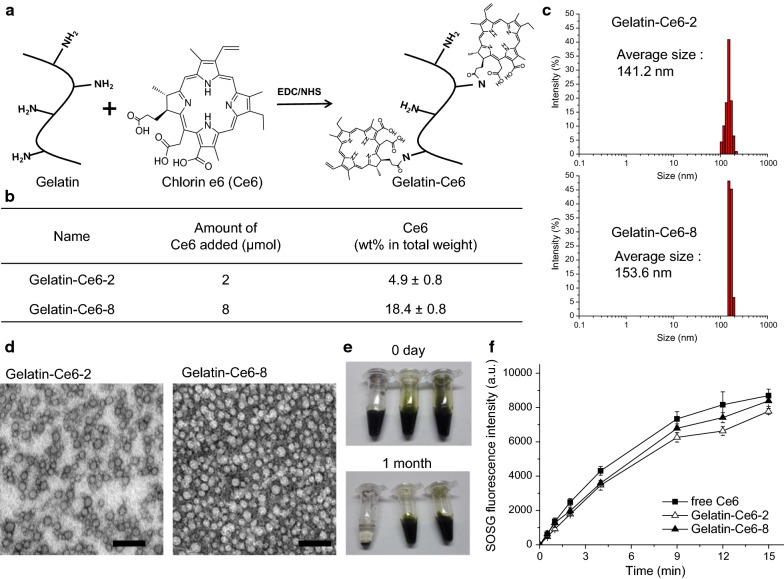
Synthesis and characterization of gelatin–conjugated chlorin e6 (gelatin–Ce6). **a** The scheme of gelatin–Ce6 synthesis. **b** The amount of gelatin–Ce6 added for synthesis and conjugated to gelatin. **c** Size distribution of gelatin–Ce6-2 and 8. **d** Transmission electron microscopy (TEM) images of gelatin–Ce6-2 and gelatin–Ce6-8. Scale bar = 100 nm. **e** The stability test of free Ce6 (left), gelatin–Ce6 (center), and gelatin–Ce6-8 (right) in PBS (pH 7.4) at 10 mg/mL Ce6. **f** Singlet oxygen generation from free Ce6, gelatin–Ce6-2, and gelatin–Ce6-8 with laser irradiation (n = 4)

Ce6 was conjugated to a gelatin polymer backbone by an amide coupling reaction between the carboxylic acid groups of Ce6 and the amine groups of gelatin (Fig. 1a). When the gelatin polymer was reacted with a 2 μmol or an 8 μmol Ce6 solution, the amounts of conjugated Ce6 were approximately 4.9% and 18.4% (wt% in total weight of the conjugates), respectively (Fig. 1b). They were named gelatin–Ce6-2 and gelatin–Ce6-8, respectively. Considering that we used gelatin polymer with 21,426 da (Additional file 1: Fig. S1), it could be calculated that about 1.8 and 8.1 Ce6 molecules were conjugated to one gelatin polymer in cased of gelatin–Ce6-2 and gelatin–Ce6-8, respectively. We observed that the sizes of gelatin–Ce6-2 and 8 were about 150 nm in aqueous condition (Fig. 1c). Their transmission electron microscopy (TEM) images significantly showed spherical structures (Fig. 1d). Therefore, we concluded that both conjugates formed self-assembled nanoparticles in aqueous condition.

Both conjugates were soluble in PBS at 10 mg/mL and remained dispersed without aggregation for more than 1 month. By contrast, free Ce6 formed aggregates within 1 day under the same conditions. Their sizes were not changed significantly after 1 month incubation showing the stability of the nanoparticles (Additional file 1: Fig. S2). Singlet oxygen generation from gelatin–Ce6 was measured using SOSG as a singlet oxygen sensor (Fig. 1d). The fluorescence intensities of SOSG in gelatin–Ce6-2 and gelatin–Ce6-8 were similar to that in free Ce6 solution. This result showed that the singlet oxygen generation of Ce6 was not reduced after conjugation to gelatin polymer, while its water solubility increased.

### Cellular uptake of gelatin–Ce6 in SCC-7 cells

**Fig. 2:**
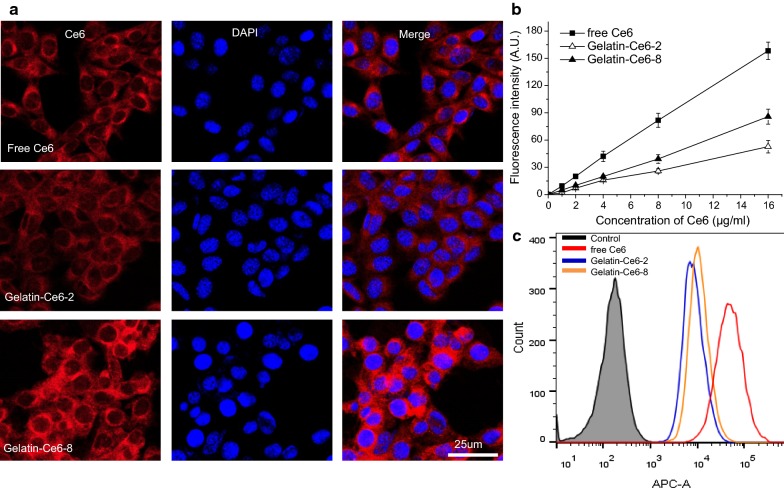
Cellular uptake of gelatin–Ce6 into SCC-7 tumor cells. **a** Confocal fluorescence microscopy images of free Ce6, gelatin–Ce6-2, and gelatin–Ce6-8 in SCC-7 cells after treatment for 2 h. The fluorescence of Ce6 was detected by Cy5.5 filter (red), and DAPI staining (blue) shows nuclei. The Ce6 concentration was 8.0 μg/mL (scale bar = 20 μm). **b** Fluorescence intensity analysis after treatment with free Ce6, gelatin–Ce6-2, and gelatin–Ce6-8 at varied concentrations of Ce6. **c** Flow cytometer data of SCC-7 cells incubated with media alone (black), free Ce6 (red), gelatin–Ce6-2 (blue), or gelatin–Ce6-8 (orange) for 2 h at a 0.2 μg/mL Ce6 concentration

Cellular uptake of free Ce6, gelatin–Ce6-2, and gelatin–Ce6-8 in SCC-7 cells was analyzed based on the fluorescence of Ce6, using fluorescence microscopy and a flow cytometer. After 2 h of incubation, free Ce6-treated SCC-7 cells had a strong red color, showing that the cells had taken up a large amount of Ce6. The tumor cells treated with gelatin–Ce6-2 and gelatin–Ce6-8 also showed a red color. However, the intensity decreased in order of free Ce6, gelatin–Ce6-8, and gelatin–Ce6-2, showing that cellular uptake increased as the amount of Ce6 relative to polymer increased. Then, we varied the concentration of each sample from 1 to 16 μg/mL of Ce6 and compared the cellular uptake (Fig. 2b). The cellular uptake of Ce6 increased with the concentration. At all concentration ranges, there was a higher cellular uptake of free Ce6 and gelatin–Ce6-8 compared to gelatin–Ce6-2. Flow cytometer data showed that free Ce6, gelatin–Ce6-8, and gelatin–Ce6-2 had similar cellular uptake, but cells treated with any of them had significantly higher intensity than untreated control cells (Fig. 2c).

### In vitro phototoxicity tests of gelatin–Ce6

**Fig. 3:**
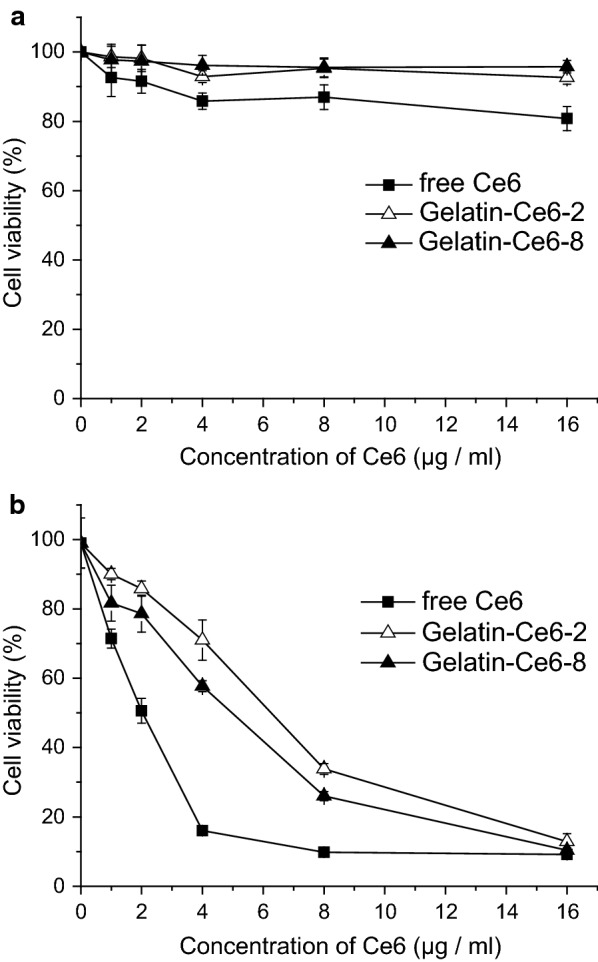
In vitro phototoxicity of gelatin–Ce6 after laser irradiation. MTT assay **a** in the dark and **b** after laser irradiation in SCC-7 cells treated with free Ce6, gelatin–Ce6-2, or gelatin–Ce6-8 for 2 h. (n = 5)

We evaluated the cytotoxicity and phototoxicity of gelatin–Ce6 in SCC-7 cells using the MTT assay. Without light, cell viability was greater than 80% with gelatin–Ce6-2 and gelatin–Ce6-8 at Ce6 concentrations up to 16 µg/mL, showing that there was no significant toxicity without irradiation (Fig. 3a). After irradiation with a 658 nm laser at 0.3 W (0.75 J), the cell viability in all samples decreased with Ce6 concentration. The phototoxicity was highest with free Ce6, and gelatin–Ce6-8 was associated with lower viability than gelatin–Ce6-2 upon irradiation. This result suggests that phototoxicity correlates with the amounts of Ce6 in the tumor cells; in other words, phototoxicity increased with cellular uptake.

### In vivo biodistribution of gelatin–Ce6 in SCC-7 tumor-bearing mice

**Fig. 4:**
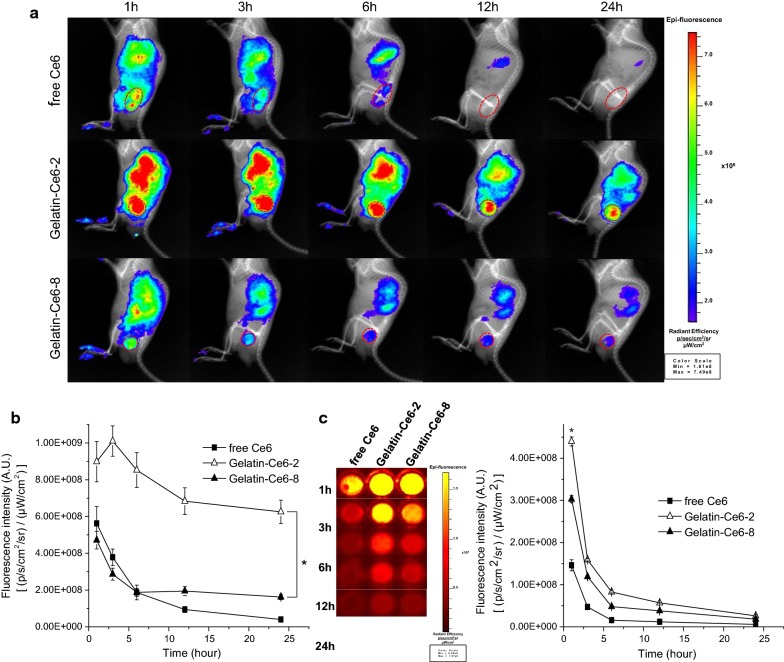
In vivo real-time near-infrared fluorescence (NIRF) imaging of gelatin–Ce6 in SCC-7 tumor-bearing mice. **a** Time-dependent whole body NIRF images of mice bearing SCC-7 tumors after intravenous injection of free Ce6, gelatin–Ce6-2, or gelatin–Ce6-8. **b** Time-dependent fluorescence intensity analysis of the tumor site according to time (n = 3). *p < 0.01. **c** NIRF images of blood samples from the mice (left) and quantification of fluorescence intensity (right). *p < 0.01

We compared the biodistribution of free Ce6, gelatin–Ce6-2, and gelatin–Ce6-8 after intravenous injection of samples (2.5 mg/kg of Ce6) into SCC-7 tumor-bearing mice. Whole body near-infrared fluorescence (NIRF) images were obtained 1, 3, 6, 12, and 24 h post-injection by an IVIS Lumina XRMS system, and they were overlaid with X-ray images [20]. In the gelatin–Ce6-2-treated mice, the NIRF signal intensity in tumor tissue was higher at all time points compared to those in the free Ce6 and gelatin–Ce6-8 groups (Fig. 4a). In the groups treated with free Ce6 or gelatin–Ce6-8, the signals in the tumor tissue decreased rapidly over time. At 24 h after intravenous injection, the average NIRF radiant efficiency of gelatin–Ce6-2 in tumor tissues was approximately 15.5 times and 3.9 times higher than that of free Ce6 and gelatin–Ce6-8, respectively (Fig. 4b). We also obtained and analyzed blood samples from the mice to investigate the blood circulation of free Ce6, gelatin–Ce6-2, and gelatin–Ce6-8. Figure 4c showed that free Ce6 and gelatin–Ce6-8 rapidly disappeared from the blood, but gelatin–Ce6-2 remained in circulation longer and was cleared more slowly.

In graph of tumor/blood ratio from all groups, the ratio of gelatin–Ce6-2 and 8 increased until 24 h, but that of free Ce6 decreased from 6 h after injection (Additional file 1: Fig. S3A). At 24 h after injection, the ratio of gelatin–Ce6-2 was more than four folds higher than that of free Ce6. In Additional file 1: Fig. S3B, we compared the intensity between tumors and bloods of gelatin–Ce6-2 and free Ce6. The ratio of blood was not changed much, but tumor ratio increased from about 1.5 to 15. These two graphs about intensity ratio proved that the high tumor accumulation of conjugates was based on the EPR effect of the conjugates due to their nanoparticle structure, not solely due to prolonged blood circulation.

### Ex vivo NIRF imaging of gelatin–Ce6

**Fig. 5:**
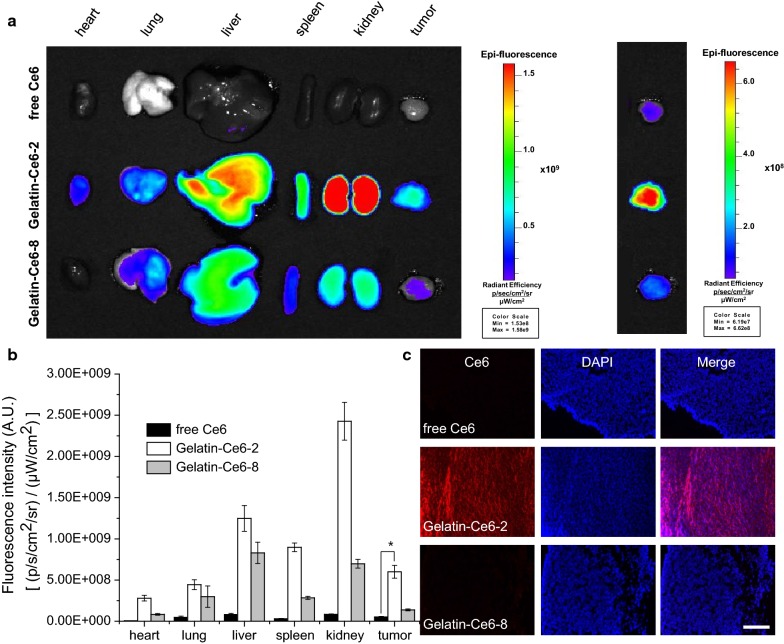
Ex vivo NIRF imaging of gelatin–Ce6 in SCC-7 tumor-bearing mice. **a** Ex vivo NIRF images of the dissected organs (heart, lung, liver, spleen, and kidney) and tumors 24 h after injection of free Ce6, gelatin–Ce6-2, or gelatin–Ce6-8 into SCC-7 tumor-bearing mice. The right image has a different intensity scale to compare tumor tissues. **b** Quantitative NIRF signal analysis of organs (heart, lung, liver, spleen, and kidney) and tumor tissue (n = 3). *p < 0.01. **c** Fluorescence images of tumor tissue slices 24 h post-injection of free Ce6, gelatin–Ce6-2, or gelatin–Ce6-8. Scale bar represents 20 μm

**Fig. 6:**
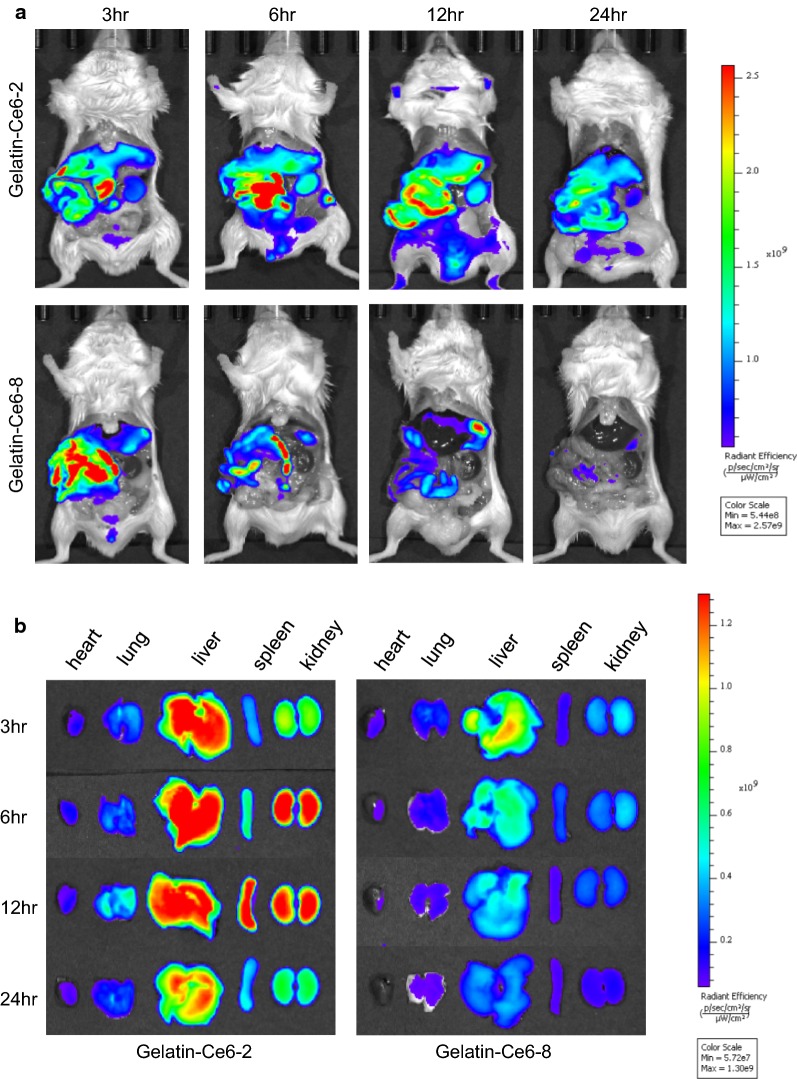
In vivo (**a**) and ex vivo (**b**) time-dependent distribution of gelatin–Ce6-2 and 8 showing their secretion pathway

Ex vivo NIRF images of tumors and major organs from the mice were obtained 24 h post-injection. Gelatin–Ce6-2-treated mice showed higher fluorescence in every organ and in tumor tissue compared with the mice treated with free Ce6 and gelatin–Ce6-8 (Fig. 5a). Gelatin–Ce6-2 showed high accumulation in the liver, spleen, and kidney, but showed approximately 11.5 and 4.4 times higher NIRF intensity in tumor tissue compared with free Ce6 and gelatin–Ce6-8, respectively (Fig. 5b). Then, the tumor tissues of each group were cryo-sectioned, and micro-scale images were obtained. NIRF signals from Ce6 were barely observable in the free Ce6- and gelatin–Ce6-8-treated groups, but the gelatin–Ce6-2-treated group showed strong signal intensity in tumor tissues (Fig. 5c). These ex vivo results showed similar trends to the in vivo imaging data. Both results demonstrated that gelatin–Ce6-2 had a longer blood circulation time and was efficiently accumulated in tumor tissue after intravenous injection. We performed animal experiments to analyze the clearance pathway of both conjugates. At 3, 6, 12, and 24 h after i.v. injection of gelatin–Ce6-2 and 8, the mice were analyzed in vivo and ex vivo (Fig. 6 and Additional file 1: Fig. S4). As shown in the data, they were secreted from body by both renal and hepatic pathway. The fluorescence intensities of the grinded organs from gel-Ce6 and 8-injected mice also showed similar trends (Additional file 1: Fig. S5).

### In vivo photodynamic therapy by gelatin–Ce6 in SCC-7 tumor-bearing mice

**Fig. 7:**
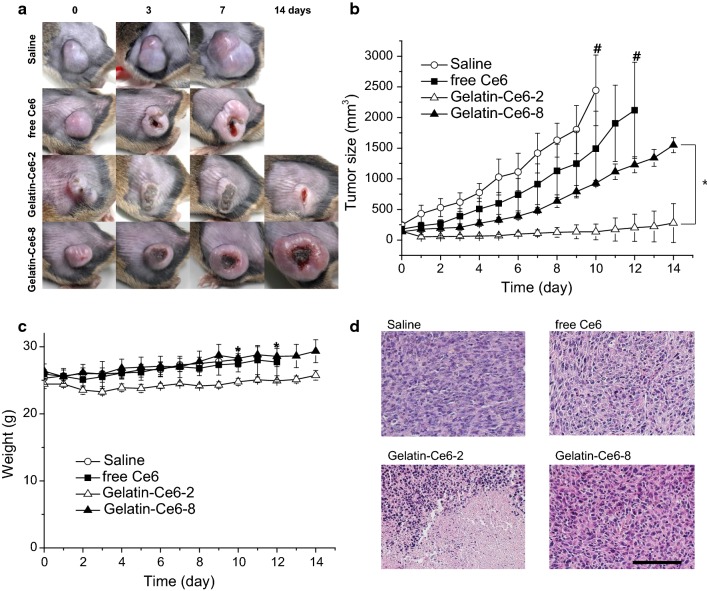
In vivo photodynamic therapy (PDT) by gelatin–Ce6 in tumor-bearing mice. **a** Tumor images after intravenous injection of saline, free Ce6, gelatin–Ce6-2, or gelatin–Ce6-8 (2.5 mg/kg of Ce6) and laser irradiation (658 nm, 0.3 W, 200 J). **b** Tumor growth after PDT by saline, free Ce6, gelatin–Ce6-2, and gelatin–Ce6-8 (2.5 mg/kg of Ce6) (n = 4). *p < 0.01. **c** Body weight change of the mice after PDT (n = 4). **d** Images of H&E-stained tumor tissues 14 days post-treatment. Scale bar represents 100 μm. ^#^Means early sacrifice due to large tumor size

**Fig. 8:**
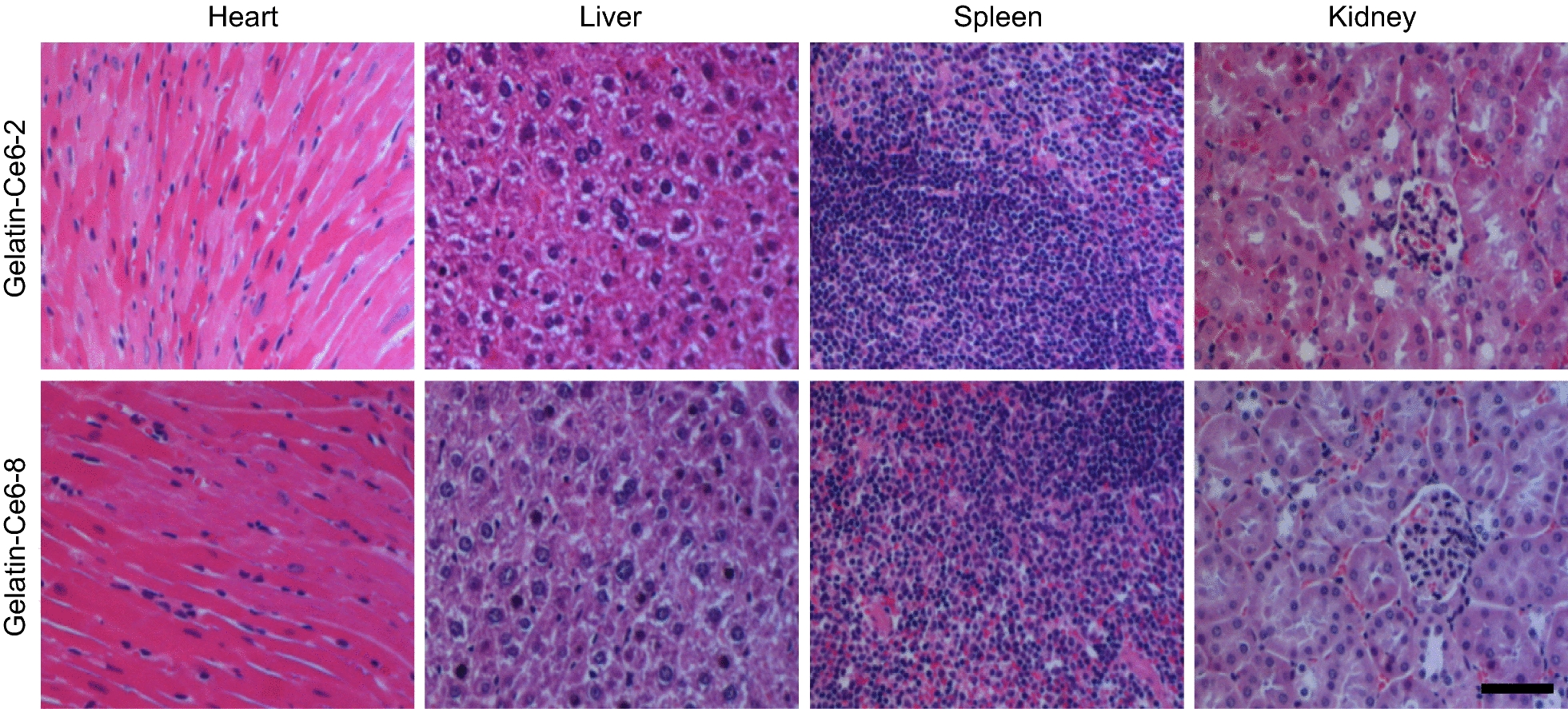
H&E stained images of the major organs after i.v. injection of gelatin–Ce6-2 and 8 into mice. Scale bar = 200 μm

To evaluate the in vivo therapeutic efficacy of free Ce6, gelatin–Ce6-2, or gelatin–Ce6-8 (2.5 mg/kg Ce6), each was injected into the SCC-7 tumor-bearing mice via the tail vein. Then, we irradiated tumor tissues with a laser (658 nm, 0.3 W, 200 J) at 4 and 12 h after injection of the samples, and tumor size was measured for up to 14 days. Three days post-injection, the laser-irradiated tumor sites in the gelatin–Ce6-2- and gelatin–Ce6-8-treated mice showed severe necrosis (Fig. 7a). After 7 days, the necrotic scar tissue was healed in the gelatin–Ce6-2-treated mice, and normal tissue began to regenerate. Compared to the saline-treated control mice after 10 days, the free-Ce6- or gelatin–Ce6-8-treated mice showed tumor volume reductions of approximately 39% and 62%, respectively (Fig. 7b). In particular, gelatin–Ce6-2-treated mice showed highly enhanced tumor reduction of 94%. The body weights of the drug-treated groups were not significantly different from the control group, indicating that there were no critical side effects (Fig. 7c). At 14 days, all mice were sacrificed, and tissues were stained with H&E to show the tissue damage and the results of treatment (Fig. 7d). The saline-treated mice did not have apoptotic or necrotic tumor cells. Incomplete tumor destruction was observed in mice treated with free Ce6 or gelatin–Ce6-8. In contrast, in the gelatin–Ce6-2-treated mice, most of the tumor tissues were destroyed by laser irradiation, and fibrosis was observed behind the epidermis. After i.v. injection of gelatin–Ce6-2 and 8 to the mice, we analyzed the major organs by H&E staining (Fig. 8). There was no significant damage in all the sliced tissue images from organs showing gelatin–Ce6 has negligible toxicity without light irradiation.

## Discussion

As we expected, conjugation of Ce6 to gelatin polymer highly increased its water solubility and stability. However, uptake into tumor cells was weakened after conjugation, as shown in comparison of fluorescence intensities after treatment with free Ce6 versus gelatin–Ce6. Toxic singlet oxygen generation correlates with the amount of photosensitizer; therefore, the post-irradiation tumor cell death also decreased with gelatin–Ce6 compared to free Ce6. This result may be because the hydrophobicity of Ce6 decreased after conjugation to gelatin. The results show that the cellular uptake of gelatin–Ce6-8 was higher than that of gelatin–Ce6-2, which contained a smaller proportion of hydrophobic Ce6. However, we think that this is not a critical problem because the enhanced tumor accumulation of gelatin–Ce6 overrides the effect of its weakened cellular uptake, as shown in the following in vivo data. Because the number of conjugated Ce6 was calculated by fluorescence per weight, there is a possibility that it was different with exact number due to quenching effect between multiple Ce6 molecules conjugated to one polymer. However, the Ce6 molecules were conjugated by amide bonds which is not degraded in short time, so that the measured number of Ce6 would be effective for photodynamic effect in the following studies.

Whole body and ex vivo imaging data showed that gelatin–Ce6 had a highly enhanced blood circulation time and tumor tissue accumulation. We think there are two reasons for this improvement. First, the increased water solubility and stability of gelatin Ce6 could prevent aggregation with serum proteins, enable escape from macrophages, and delay its removal by the immune system. This hypothesis is supported by the in vivo data that gelatin–Ce6-2 showed longer circulation time in blood and higher accumulation in tumor tissue after intravenous injection. Large portion of Ce6 in gelatin–Ce6-8 increased hydrophobicity which might result in relatively increased binding to serum proteins and accelerated their removal from blood compared to gelatin–Ce6-2. As reported by Lyon et al., hydrophobicity of the molecules increases plasma clearance and reduced therapeutic index [21]. Second, the increased molecular weight after conjugation with a large gelatin polymer also resulted in high accumulation through EPR, as described above [12]. These factors promoted efficient delivery of Ce6 to the target tumor tissue and increased the therapeutic efficacy of gelatin–Ce6 after laser irradiation. On the other hand, the prolonged blood circulation of gelatin–Ce6 also resulted in higher accumulation in normal tissues. However, the enhanced signals were mainly observed in kidney, liver, and spleen, which are major excretory organs [22]. This means that the injected gelatin–Ce6 would be maintained in the body for a longer time. We think that this uptake by these organs would not cause significant harm because the organs are located inside the body and blocked from light, preventing any photodynamic effect. In addition, it needs to be considered that the optical imaging-based biodistribution might not truly reflect the real one due to signal loss during tissue penetration or quenching. If possible, imaging with radioisotope will provide more reliable information.

There have been a large variety of biomaterials created for drug delivery, but only a few of them have been approved by the FDA and used in clinical practice [23]. Minimizing the chances of cytotoxicity and receiving FDA approval for human usage are highly important in this research. Our material, gelatin–Ce6, is composed of only two components: gelatin, a widely used biopolymer, and Ce6 as the photosensitizer. Importantly, with this simple structure, we achieved highly increased tumor accumulation and enhanced therapeutic result in vivo [24]. Recent trend is that more complex nanoparticles are developed for further improved drug delivery. However, Daniel et al. pointed out that the increased complexity may also increase cost and difficulty for large scale production for clinical application [25]. In this point of view, we propose that gelatin–Ce6 is more suitable for FDA approval and clinical application compared to other, more complicated structures with many components. In addition, gelatin will be degraded and excreted from the body after a long time, which will further decrease potential risk [26].

## Conclusions

In summary, we developed a gelatin–Ce6 polymer conjugate for efficient PDT of cancer in vivo. Gelatin–Ce6 was synthesized by chemically conjugating multiple Ce6 molecules to a gelatin polymer. To determine the optimal chemical structure for PDT in vivo, we prepared two types of gelatin–Ce6: gelatin–Ce6-2 and gelatin–Ce6-8. These conjugates had high solubility in water and were stable for a long time, unlike Ce6, which rapidly aggregated at the same concentration. Gelatin–Ce6 was non-toxic in the absence of light, but generated singlet oxygen and killed tumor cells upon laser irradiation. In vivo tumor accumulation of gelatin–Ce6-2 was much higher than that of free Ce6 or gelatin–Ce6-8 after intravenous injection into mice. After laser irradiation to the tumor site, gelatin–Ce6-2 showed superior tumor suppression, indicating an enhanced PDT effect. These positive results in vivo may be due to the longer blood circulation and higher tumor accumulation of gelatin–Ce6 by EPR. These overall results demonstrate the potential of gelatin–Ce6 as an effective therapeutic agent for PDT against various cancers. In particular, the simple structure of gelatin–Ce6, which is only composed of biopolymer gelatin and photosensitizer, will also be advantageous for clinical application and commercialization.

## Additional file

**Additional file 1.** Additional figures.

## Abbreviations

PDT: photodynamic therapy
Ce6: chlorin e6
EPR: enhanced permeation and retention
MTT: 3-[4,5-dimethyl-thiazol-2-yl]-2,5-diphenyltetrazolium bromide
SOSG: Singlet Oxygen Sensor Green Reagent
NIRF: near-infrared fluorescence

## References

[1] Dolmans DEJGJ, Fukumura D, Jain RK (2003). Photodynamic therapy for cancer. Nat Rev Cancer.

[2] Lovell JF, Liu TWB, Chen J, Zheng G (2010). Activatable photosensitizers for imaging and therapy. Chem Rev.

[3] Celli JP, Spring BQ, Rizvi I, Evans CL, Samkoe KS, Verma S, Pogue BW, Hasan T (2010). Imaging and photodynamic therapy: mechanisms, monitoring, and optimization. Chem Rev.

[4] Moore CM, Pendse D, Emberton M (2009). Photodynamic therapy for prostate cancer-a review of current status and future promise. Nat Clin Pract Urol.

[5] Park W, Cho S, Han J, Shin H, Na K, Lee B, Kim D-H (2018). Advanced smart-photosensitizers for more effective cancer treatment. Biomater Sci.

[6] Tada DB, Baptista MS (2015). Photosensitizing nanoparticles and the modulation of ROS generation. Front Chem.

[7] Lee Y-H, Ma Y-T (2017). Synthesis, characterization, and biological verification of anti-HER2 indocyanine green-doxorubicin-loaded polyethyleneimine-coated perfluorocarbon double nanoemulsions for targeted photochemotherapy of breast cancer cells. J Nanobiotechnol.

[8] Yi G, Hong SH, Son J, Yoo J, Park C, Choi Y, Koo H (2018). Recent advances in nanoparticle carriers for photodynamic therapy. Quant Imaging Med Surg.

[9] Göke K, Lorenz T, Repanas A, Schneider F, Steiner D, Baumann K, Bunjes H, Dietzel A, Finke JH, Glasmacher B, Kwade A (2018). Novel strategies for the formulation and processing of poorly water-soluble drugs. Eur J Pharm Biopharm.

[10] van Dongen GAMS, Visser GWM, Vrouenraets MB (2004). Photosensitizer-antibody conjugates for detection and therapy of cancer. Adv Drug Deliv Rev.

[11] Kim KS, Kim J, Kim DH, Hwang HS, Na K (2018). Multifunctional trastuzumab-chlorin e6 conjugate for the treatment of HER2-positive human breast cancer. Biomater Sci.

[12] Maeda H, Wu J, Sawa T, Matsumura Y, Hori K (2000). Tumor vascular permeability and the EPR effect in macromolecular therapeutics: a review. J Control Release.

[13] Maeda H (2010). Tumor-selective delivery of macromolecular drugs via the EPR Effect: background and future prospects. Bioconjug Chem.

[14] Fox ME, Szoka FC, Fréchet JMJ (2009). Soluble polymer carriers for the treatment of cancer: the importance of molecular architecture. Acc Chem Res.

[15] Duncan R (2006). Polymer conjugates as anticancer nanomedicines. Nat Rev Cancer.

[16] Olsen D, Yang C, Bodo M, Chang R, Leigh S, Baez J, Carmichael D, Perälä M, Hämäläinen E-R, Jarvinen M, Polarek J (2003). Recombinant collagen and gelatin for drug delivery. Adv Drug Deliv Rev.

[17] Chen Q, Cao L, Wang J, Jiang L, Zhao H, Yishake M, Ma Y, Zhou H, Lin H, Dong J, Fan Z (2018). Bioinspired modification of poly(l-lactic acid)/nano-sized beta-tricalcium phosphate composites with gelatin/hydroxyapatite coating for enhanced osteointegration and osteogenesis. J Biomed Nanotechnol.

[18] Lee SJ, Koo H, Jeong H, Huh MS, Choi Y, Jeong SY, Byun Y, Choi K, Kim K, Kwon IC (2011). Comparative study of photosensitizer loaded and conjugated glycol chitosan nanoparticles for cancer therapy. J Control Release.

[19] Koo H, Lee S, Na JH, Kim SH, Hahn SK, Choi K, Kwon IC, Jeong SY, Kim K (2012). Bioorthogonal copper-free click chemistry in vivo for tumor-targeted delivery of nanoparticles. Angew Chem Int Ed.

[20] Son J, Yang SM, Yi G, Roh YJ, Park H, Park JM, Choi M-G, Koo H (2018). Folate-modified PLGA nanoparticles for tumor-targeted delivery of pheophorbide a in vivo. Biochem Biophys Res Commun.

[21] Lyon RP, Bovee TD, Doronina SO, Burke PJ, Hunter JH, Neff-LaFord HD, Jonas M, Anderson ME, Setter JR, Senter PD (2015). Reducing hydrophobicity of homogeneous antibody-drug conjugates improves pharmacokinetics and therapeutic index. Nat Biotech.

[22] Masereeuw R, Russel FGM (2001). Mechanisms and clinical implications of renal drug excretion. Drug Metab Rev.

[23] Aaron CA, Balabhaskar P, Kapil P, Samir M (2017). Clinical and commercial translation of advanced polymeric nanoparticle systems: opportunities and material challenges. Transl Mater Res.

[24] Cheng Z, Al Zaki A, Hui JZ, Muzykantov VR, Tsourkas A (2012). Multifunctional nanoparticles: cost versus benefit of adding targeting and imaging capabilities. Science.

[25] Bobo D, Robinson KJ, Islam J, Thurecht KJ, Corrie SR (2016). Nanoparticle-based medicines: a review of FDA-approved materials and clinical trials to date. Pharm Res.

[26] Tondera C, Hauser S, Krüger-Genge A, Jung F, Neffe AT, Lendlein A, Klopfleisch R, Steinbach J, Neuber C, Pietzsch J (2016). Gelatin-based hydrogel degradation and tissue interaction in vivo: insights from multimodal preclinical imaging in immunocompetent nude mice. Theranostics.

