# Supplementary material for: Gelatin–chlorin e6 conjugate for in vivo photodynamic therapy
Source: J Nanobiotechnology. 2019 Apr 5;17:50. doi: 10.1186/s12951-019-0475-1 (PMC6449946; doi:10.1186/s12951-019-0475-1)
Supplement: Supplementary file 1 — Additional file 1. Additional figures. [file 12951_2019_475_MOESM1_ESM.doc]

**Additional information**

**Gelatin-chlorin e6 conjugate for *in vivo* photodynamic therapy**

Jihwan Son^1,2^, Gawon Yi^1,2^, [Moon-Hwa Kwak](https://www.researchgate.net/scientific-contributions/2017443683_Moon-Hwa_Kwak)^3^, Seung Mok Yang^2,3^, Jae Myung Park^3,4^, Bo-In Lee^3,4^, Myung-Gyu Choi^3,4^, Heebeom Koo^1,2,4*^

^1^ Department of Medical Life Sciences**,** College of Medicine, The Catholic University of Korea, 222 Banpo-daero, Seocho-gu, Seoul, 06591, Republic of Korea

Full list of author information is available at the end of the article

* Correspondence: hbkoo@catholic.ac.kr

**Fig. S1.** Gel permeation chromatography (GPC) data showing the molecular weight of gelatin polymer used.

**Fig. S2.** Sizes of gelatin-Ce6-2 and 8 after one month incubation at room temperature.

**Fig. S3.** Fluorescence intensity ratio of the tumors and bloods from free Ce6, gelatin-Ce6-2, and 8-injected mice. (a) Tumor to blood ratio of the three groups. (b) Tumor and blood ratio comparing free Ce6 and gelatin-Ce6-2 group.

**Fig. S4.** Quantitative data of the time-dependent fluorescence intensity in Fig. 6B

**Fig. S5.** Fluorescence images of the grinded solutions of major organs in Fig. 6B after i.v. injection of gelatin-Ce6-2 and 8.
